# Association of Ion Concentration with Immune-Related Adverse Events and Prognosis in Lung Cancer Patients Treated with PD-1/PD-L1 Inhibitors

**DOI:** 10.7150/jca.120666

**Published:** 2026-01-01

**Authors:** Chen-Wei Liao, Juan Chen, Jia-Si Liu, Lei She, Ting Zou, Ya Wang, Zhan Wang, Zhao-Qian Liu

**Affiliations:** 1 Xiangya School of Pharmacy, Central South University, Changsha 410078; P. R. China.; 2 Department of Pharmacy, Xiangya Hospital, Central South University, Changsha 410078; P. R. China.; 3 Lung Cancer and Gastrointestinal Unit, Department of Medical Oncology, Hunan Cancer Hospital/The Affiliated Cancer Hospital of Xiangya School of Medicine, Central South University, Changsha 410031, P. R. China.; 4 Department of Clinical Pharmacology, Hunan Key Laboratory of Pharmacogenetics, and National Clinical Research Center for Geriatric Disorders, Xiangya Hospital, Central South University, Changsha 410008, P. R. China.; 5 National Institution of Drug Clinical Trial, Xiangya Hospital, Central South University, Changsha 410008, P. R. China.; 6 The Hunan Institute of Pharmacy Practice and Clinical Research, Changsha 410008, P. R. China.; 7 Department of Pharmacy, The Second Affiliated Hospital, Zhejiang University School of Medicine, Hangzhou, 310009, P. R. China.

**Keywords:** lung cancer, ICIs, irAEs, ion concentration, PFS, biomarkers

## Abstract

**Objectives**: irAEs were associated with immunotherapy response in cancer treatment, but severe irAEs discontinued immunotherapy and affected the quality of life. This study aimed to identify ion concentrations as potential biomarkers for irAEs and prognosis in lung cancer patients receiving ICI therapy.

**Methods:** A retrospective analysis was conducted on 459 lung cancer patients who received ICI treatment at Xiangya Hospital from April 2019 to May 2023. Patient characteristics, ion concentrations (K^+^, Na^+^, Cl^-^, Ca^2+^, PO_4_^3-^ and Mg^2+^), irAEs, and prognosis were systematically collected. Univariable and multivariable regression analyses, including binary logistic regression and Cox regression models, were employed to identify factors associated with irAEs and PFS.

**Results:** Among 459 lung cancer patients receiving ICI treatment, 378 (82.4%) of the patients suffered irAEs. PD-L1 expression, ICI cycles, ORR and DCR were linked to irAEs occurrence. Cardiotoxicity, hypothyroidism, and dermatoxicity were the predominant irAEs types, but mostly mild to moderate. Notably, elevated potassium (K^+^) level was significantly correlated with both a higher risk of irAEs and longer PFS.

**Conclusions:** The findings suggest that K^+^ concentration prior to initiating treatment with ICIs may be a biomarker of irAEs and PFS in lung cancer patients.

## Introduction

Lung cancer ranks among the most common and lethal malignancies worldwide 1. Most patients present with distant metastases at diagnosis, substantially complicating treatment 2. Immunotherapy, especially immune checkpoint inhibitors (ICIs), has significantly improved clinical prognosis 3, 4. By activating cytotoxic T lymphocytes against tumors, ICIs yield considerable clinical benefits; however, this broad immune activation also leads to immune-related adverse events (irAEs) in 60-80% of patients, most commonly affecting the skin, gastrointestinal tract, and endocrine systems 5-7. For instance, immune-mediated diarrhea and colitis often occur 4 to 6 weeks after treatment initiation 8. As immunotherapy is widely used to identify biomarkers and risk factors for predicting toxicity has become increasingly important 9-12.

Ions include potassium (K^+^), sodium (Na^+^), magnesium (Mg^2+^), calcium (Ca^2+^), chloride (Cl^-^), and phosphate (PO_4_^3-^) which play vital roles in transcellular and intracellular signaling. These signaling processes are essential for immune activation and immunological memory 13-15. Maintaining ionic balance is critical for cellular function and systemic physiological stability; its disruption may affect the tumor microenvironment (TME) 14, 16. Within the TME, competition between T cells and tumor cells for essential ions can interfere with metabolic reprogramming and impair T cell-mediated antitumor responses 17, 18. T cells, central to antitumor immunity, are activated upon T cell receptor (TCR) recognition of tumor antigen peptides presented by major histocompatibility complex (MHC) molecules 19. Key T cell subsets, including cytotoxic T lymphocytes (CTLs), helper T cells (Th), and regulatory T cells (Tregs), orchestrate immune responses in the TME, and their interplay critically shapes the outcome of immunotherapy 20. Consequently, ionic imbalance within the TME may substantially influence T cell function and thereby affect both the safety and efficacy of ICIs 21. Emerging evidence suggests that metal ion-modulated immunotherapy represents a promising therapeutic strategy, with ions participating in early immune regulation 22, indicating that plasma ion levels may be linked to immunotherapy response and irAEs.

Therefore, this retrospective study aimed to evaluate the association between ion concentrations and the occurrence of irAEs as well as prognosis in lung cancer patients treated with ICIs.

## Materials and Methods

### Patient collection

The study population included 459 patients with histologically or cytologically confirmed diagnosis of lung cancer treated with immune checkpoint inhibitors at Xiangya Hospital from April 2019 to May 2023. The ethics committee of Xiangya Hospital, Central South University approved this retrospective study (2022100970), and all patients have provided written informed consent.

### Study assessments

Adverse events were graded using the National Cancer Institute Common Terminology Criteria for Adverse Events (CTCAE) version 5.0. Treatment responses were assessed using the Immune Response Evaluation Criteria in Solid Tumors (iRECIST), with the objective response rate (ORR) defined as the proportion of patients achieving a confirmed complete or partial response (CR/PR) sustained for ≥ 4 weeks. The disease control rate (DCR) was defined as the proportion of patients with the best overall response of CR, PR, or stable disease (SD) lasting ≥ 12 weeks. Progression-free survival (PFS) was defined as the time from immunotherapy initiation until radiologically confirmed disease progression per iRECIST, death from any cause, or the last valid tumor assessment date for censored observations.

### Treatment and data collection

The clinical characteristics of patients were systematically collected, including age, ICIs treatment cycle, gender, smoking history, tumor histology, disease stage, immune checkpoint inhibitor type, metastasis status, treatment line, preexisting diseases, PD-L1 expression, driver mutation status, development of immune-related adverse events (irAEs). The clinical characteristics of the patients were summarized in Table 1. The ion concentration (K^+^, Na^+^, Cl^-^, Ca^2+^, PO_4_^3-^ and Mg^2+^) of the enrolled patients before the first application of immune checkpoint inhibitors were obtained by the medical records.

### Statistical analysis

Categorical variables were reported as frequencies and percentages, while continuous variables were presented as means ± SD for normal distributions or medians (IQR) for skewed ones. Chi-square tests compared categorical variables, independent t-tests assessed normally distributed continuous variables, and the Mann-Whitney U-test was used for skewed ones. Binary logistic regression identified factors associated with irAEs. Cox regression models determined PFS-associated factors, with significant univariable variables entered into multivariable models. Cut-off values of high and low concentration of the ions for analyses of irAEs, ORR, and DCR were determined using ROC curves and Youden's index. The optimal PFS critical value was set using the surv_cutpoint() function in the survminer package. All analyses used SPSS 26.0 and R 4.1.1, with* P*< 0.05 indicating significance.

## Results

### Clinical characteristics of the patients

A total of 459 lung cancer patients who received immunotherapy were recruited in this study (Table 1). The irAEs occurred in 378 patients (82.4%). The median age of patients who developed irAEs were 61.1 ± 8.4 years compared with 60.2 ± 8.1 years among those who did not. Gender distribution was comparable (female [13.2%] and male [86.8%] with irAEs) vs (female [14.8%] and male [85.2%] without irAEs). The development of irAEs was significantly associated with a high expression of programmed death-ligand 1 (PD-L1) (*P = 0.005*), number of ICI treatment cycles (*P = 0.007*), objective response rate (ORR, *P = 0.042*), and disease control rate (DCR, *P = 0.008*). In contrast, no significant associations were observed between irAEs and gender, age, tumor histology, disease stage, smoking history, type of ICI, line of treatment, preexisting diseases, or driver mutation status.

### irAEs outcomes

**Figure 1:**
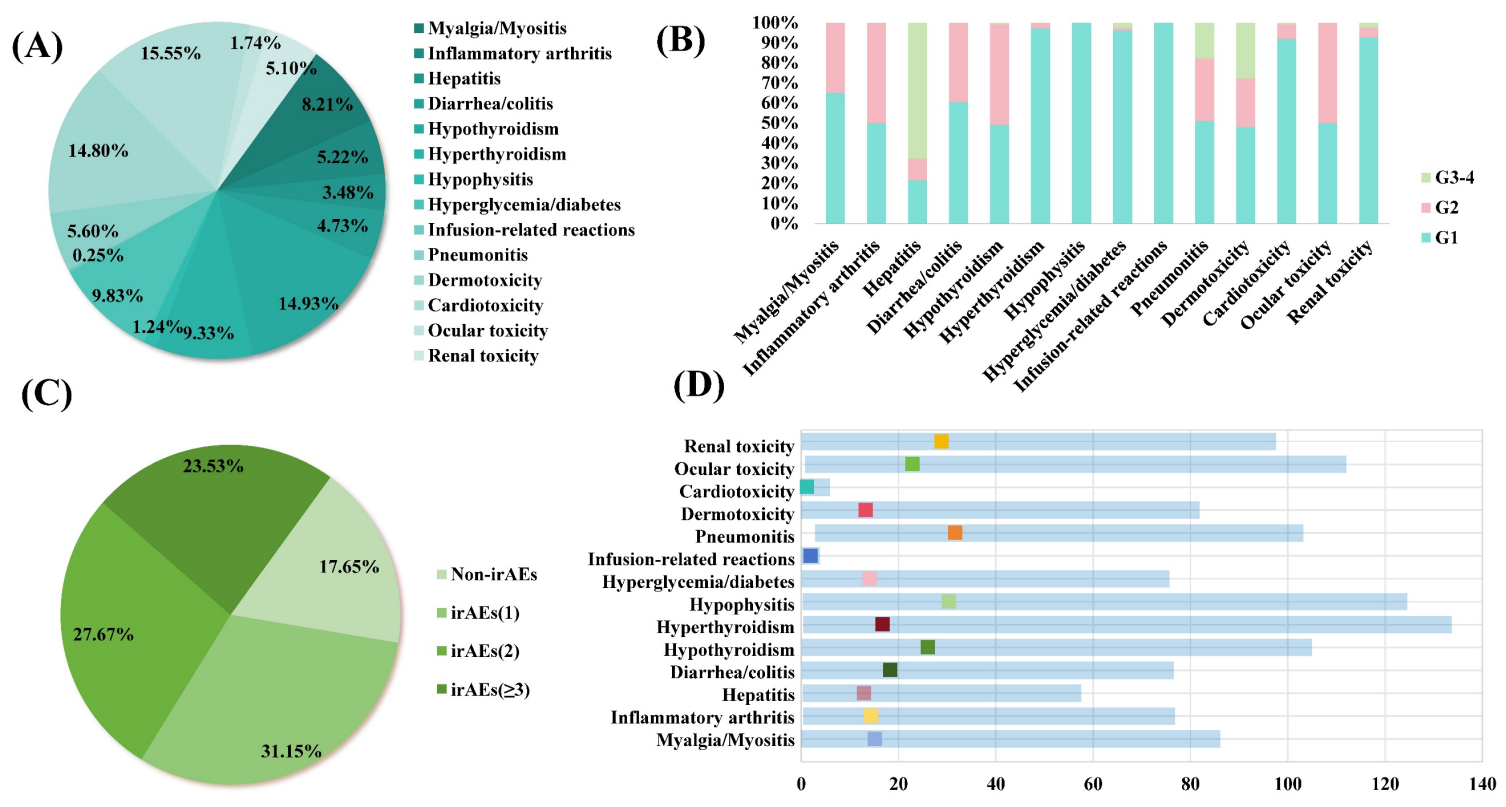
irAEs in lung cancer patients receiving ICIs. (A)The categories and proportions of irAEs. (B)The incidence of irAEs. (C) The proportions single or multiple irAEs. (D) Time to onset of irAEs.

The characteristics of irAEs are shown in Figure 1. The most prevalent immune-related adverse events (irAEs) were cardiotoxicity (15.55%), followed by hypothyroidism (14.93%), and dermotoxicity (14.80%) (Figure 1A). During the follow-up period, endocrine toxicity, musculoskeletal toxicity, and cardiotoxicity predominantly exhibited Grade 1-2 irAEs, while dermotoxicity and hepatitis had a higher incidence of high-grade (Grade 3-4) irAEs. Notably, most irAEs were mild to moderate in severity (Figure 1B). The majority of patients experienced either one or two types of irAEs (Figure 1C). The time of onset of each irAEs was different. Pneumonitis had the longest median onset of 31.73(2.87-103.16) weeks compared with other toxicities. However, Cardiotoxicity with a median onset of 1.27(0.01-5.97) weeks, indicating a relatively rapid onset compared to other irAEs (Figure 1D).

### The association of ion concentrations with irAEs and treatment response

The ROC curve was used to determine the optimal cut-off values of ion concentrations (K^+^, Na^+^, Cl^-^, Ca^2+^, PO_4_^3-^ and Mg^2+^) for irAEs, ORR and DCR (Table S1). K^+^ concentration showed significant associations with irAEs, ORR, and DCR. A higher pre-treatment K^+^ concentration was significantly associated with an increased incidence of irAEs (76.46%; *P = 0.022*). It was also associated with improved ORR (56.97%; *P = 0.028*) and DCR (71.57%; *P = 0.008*), suggesting a potential role for K^+^ levels in predicting both toxicity and treatment response. Additionally, Cl^-^ concentration showed a significant correlation with the occurrence of irAEs. Furthermore, Ca^2+^ concentration was associated with DCR, indicating a possible impact on treatment efficacy. In contrast, the lack of statistical significance in the association between Na^+^, PO_4_^3-^, and Mg^2+^ concentrations and each clinical index may be attributed to insufficient sample size or the weak direct role of these ions in immune regulation (*P* > 0.05).

### K^+^ concentration associated with irAEs

Univariable and multivariable regression analyses were performed to evaluate the association between potassium (K⁺) concentration and immune-related adverse events (irAEs) (Table 2). In the univariable analysis, a higher K⁺ level was significantly associated with an increased risk of irAEs (OR = 1.81, 95% CI: 1.08-3.01, *P = 0.023*). This association remained significant after multivariable adjustment (OR = 1.86, 95% CI: 1.10-3.11, *P = 0.019*). These results suggest that elevated K⁺ levels are a potential risk factor for irAEs.

### K^+^ concentration associated with PFS

**Figure 3:**
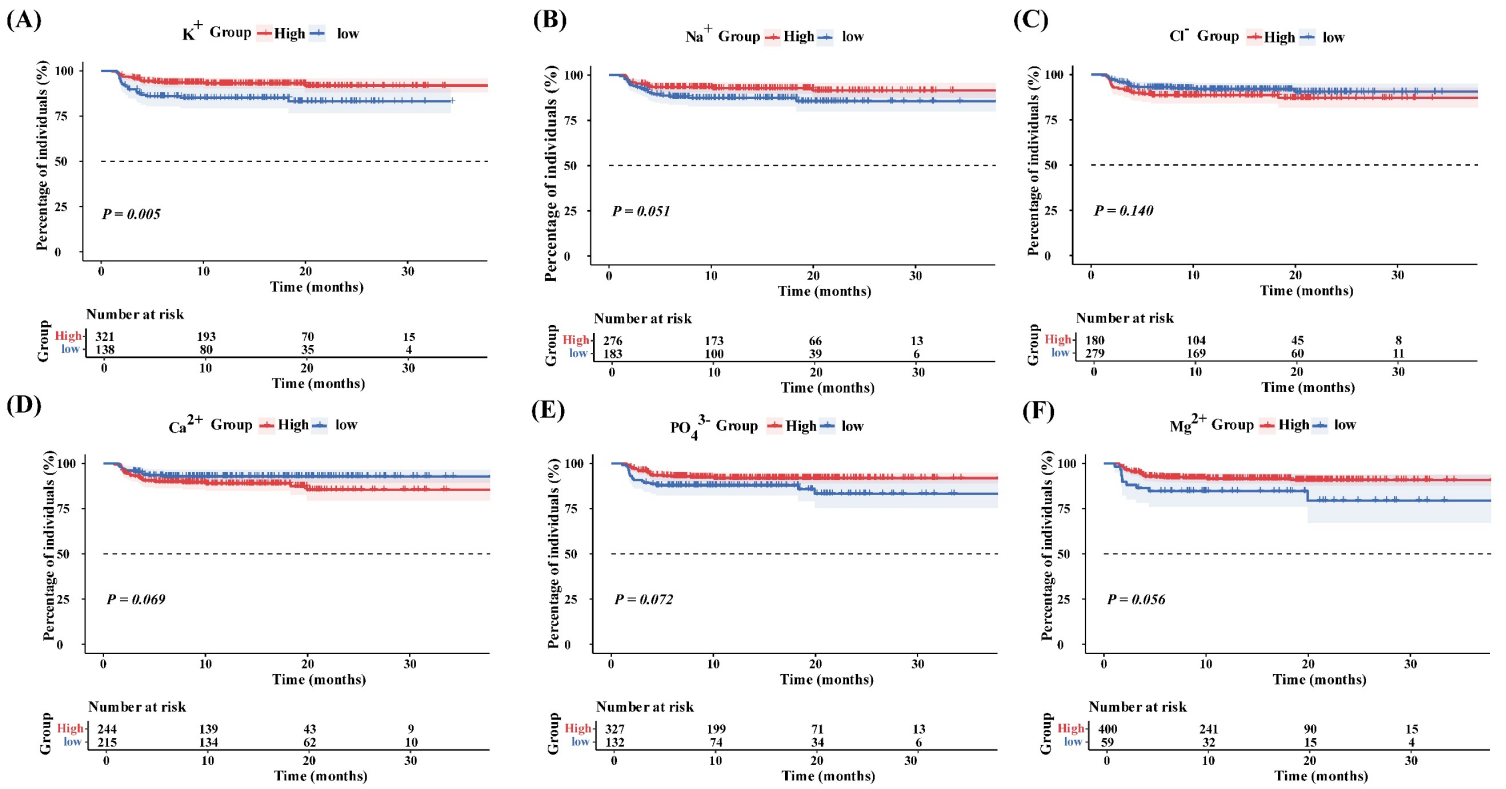
Progression-free survival stratified by serum ion concentrations. (A) High vs. low potassium ions. (B) High vs. low sodium ions. (C) High vs. low chloride ions. (D) High vs. low calcium ions. (E) High vs. low phosphate ions. (F) High vs. low magnesium ions.

The optimal cut-off values for each ion concentration (K^+^, Na^+^, Cl^-^, Ca^2+^, PO_4_^3-^ and Mg^2+^) were obtained by using the surv_cutpoint() function in the survminer package of R 4.1.1 software (Table S1). The Kaplan - Meier curve showed that the survival probability was higher in the high K^+^ group than in the low K^+^ group. In contrast, no significant differences in survival were observed for Na^+^, Cl^-^, Ca^2+^, PO_4_^3-^ and Mg^2+^ (*P > 0.05*) (Figure 3). Univariable analysis revealed a significantly lower outcome risk in the high K^+^ group (HR = 0.43, 95% CI: 0.24 -0.79, *P* = 0.006). multivariable analysis confirmed that a high K^+^ level was an independent prognostic factor for prolonged progression-free survival (HR = 0.52, 95% CI: 0.28 -0.96, *P* = 0.036) (Table 3).

## Discussion

Although immune checkpoint inhibitors (ICIs) have significantly advanced cancer treatment, the absence of reliable biomarkers for precise patient selection remains a major challenge 23, 24. Investigating the relationship between immune-related adverse events (irAEs) and prognostic biomarkers may provide new insights. While multiple studies have confirmed a positive correlation between the occurrence of irAEs and improved prognosis in cancer patients 25-27, commonly shared biomarkers for both irAEs and prognosis remain scarce. In this study, we found that elevated potassium ion (K^+^) concentration was associated with higher objective response rate (ORR) and disease control rate (DCR). Moreover, patients with higher K^+^ levels were more prone to developing irAEs and exhibited better prognoses.

As previously reported, factors such as advanced age, gender, and smoking history have been suggested to correlate with irAE development 28-30. However, the present study did not identify significant associations between irAEs and demographic characteristics, tumor histology, or comorbidities, implying that immune-related factors may play a more dominant role in determining susceptibility to irAEs. Our findings align with earlier reports indicating that the incidence of irAEs is linked to PD-L1 expression, number of treatment cycles, and clinical outcomes such as ORR and DCR in ICI-treated patients 31-33.

The overall incidence of irAEs in our cohort was 82.4%, consistent with previously reported rates following ICI therapy 34. Although the incidence of grade ≥ 3 irAEs is generally around 23% in literature, with most events being low-grade and self-limiting 35, our study observed a comparatively higher rate of severe (grade ≥3) irAEs. This discrepancy may be partly explained by a broader patient population, as prospective studies such as that by Fujimoto *et al.* have demonstrated that patients ineligible for clinical trials tend to experience higher incidences of grade ≥3 adverse events 36.

Mechanistically, potassium ions (K^+^) are essential for electrochemical regulation, cellular homeostasis, and metabolic signaling. Elevated extracellular K^+^ concentrations in the tumor microenvironment disrupt the intracellular K^+^ gradient, which in turn inhibits voltage-gated Kv1.3 channels and blocks the Akt-mTOR signaling pathway. These disruptions induce metabolic alterations and promote epigenetic modifications, ultimately leading to T-cell exhaustion 37-39. Moreover, intratumoral high K^+^ regulates the polarization of tumor-associated macrophages (TAMs) toward an immunosuppressive phenotype via the inward rectifier K⁺ channel Kir2.1, and K⁺ released from necrotic tumor cells creates a vicious cycle that suppresses CD8⁺ T-cell function 40, 41. Paradoxically, such potassium overload also directly restricts tumor growth by inducing tumor cell apoptosis and stabilizing G-quadruplex structures in the promoter regions of oncogenes such as c-Myc, thereby repressing their transcription and inhibiting cellular proliferation 42, 43. Consequently, Elevated K^+^ serves as a biomarker of tumor cell death and systemic immune dysregulation, wherein released K^+^ and antigens prime T cells improving tumor control and prognosis while increasing irAE risk.

However, the study has some limitations, the retrospective design and single- center cohort resulted in limited generalizability of the results, and unmeasured confounders such as comorbid medications may interfere with electrolyte levels. Therefore, prospective studies are urgently needed to further validate causality and establish the reliability of K^+^ as a predictive tool.

## Conclusions

In conclusion, this study highlights the importance of K^+^ monitoring in the management of ICI-treated patients. As a potential biomarker for irAEs and prognosis, K^+^ provides a theoretical basis for identifying patients at high risk of irAEs and optimizing monitoring protocols, which is expected to improve clinical outcomes in lung cancer immunotherapy.

## Supplementary Material

Supplementary table.

## Figures and Tables

**Figure 2:**
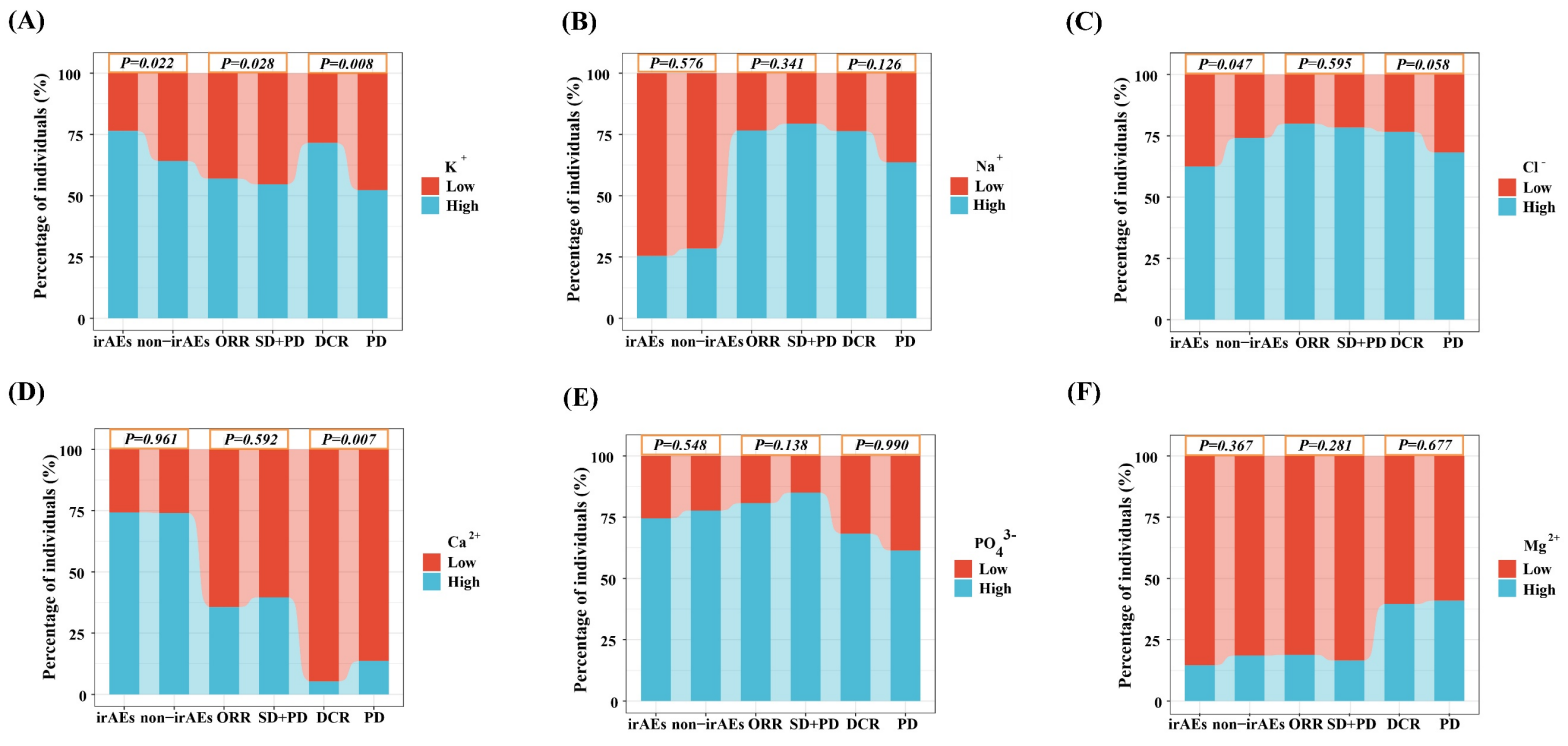
Proportions of patients with irAEs, ORR, and DCR, stratified by serum ion levels. (A) Potassium. (B) Sodium. (C) Chloride. (D) Calcium. (E) Phosphate. (F) Magnesium.

**Table 1:** Patient characteristics of 459 lung cancer patients in this study.

Characteristics	No. of patients (N=459)		
	Non-irAEs (%) (N=81)	irAEs (%) (N=378)	*P*
Age			0.371
Mean ± SD	60.2±8.1	61.1±8.4	
<60	41 (50.6)	155 (41.0)	0.112
≥60	40 (49.4)	223 (59.0)	
ICIs treatment cycle	6.0 (4.0, 9.2)	8.0 (4.0, 14.0)	0.007
Gender			
Female	12 (14.8)	50 (13.2)	0.704
Male	69 (85.2)	328 (86.8)	
Histology			0.880
Adenocarcinoma	28 (34.6)	134 (35.4)	
Non-Adenocarcinoma	53 (65.4)	244 (64.6)	
Stage			0.464
I/I	4 (4.9)	10 (2.6)	
III/IV	77 (95.1)	368 (97.4)	
Smoking status			
Former/current	63 (77.8)	298 (78.8)	0.833
Never	18 (22.2)	80 (21.2)	
Type of ICI			0.660
PD-1	69 (85.2)	333 (88.1)	
PD-L1	10 (12.3)	40 (10.6)	
PD-1/PD-L1	2 (2.5)	5 (1.3)	
Distant metastasis			0.129
Yes	43 (53.1)	235 (62.2)	
No	38 (46.91)	143 (37.8)	
No. of Treatment line			0.459
1	70 (86.4)	314 (83.1)	
≥2	11 (13.6)	64 (16.9)	
Preexisting diseases			0.549
Yes	57 (70.4)	253 (66.9)	
No	24 (29.6)	125 (33.1)	
PD-L1 expression%			0.005
<1	9 /37(24.3)	70/169 (41.4)	
1-49	8 /37(21.6)	54/169 (32.0)	
>50	20/37 (54.1)	45 /169(26.6)	
EGFR mutation			0.147
Present	5/41 (12.2)	38/170 (22.4)	
Absent	36/41 (87.8)	132/170(77.6)	
ALK mutation			0.616
Present	1/43 (2.4)	2/153 (1.3)	
Absent	41 /43(97.6)	151/153(98.7)	
KRAS mutation			0.272
Present	3/27 (11.1)	34 /170(20.0)	
Absent	24/27 (88.9)	136/170(80.0)	
MET mutation			0.279
Present	0/34 (0.0)	5/149 (3.4)	
Absent	34 /34(100.0)	144/149(96.6)	
BRAF mutation			0.196
Present	0 /36(0.0)	6/134 (4.5)	
Absent	36/36 (100.0)	128/134(95.5)	
Best Response			0.037
CR	1 (1.2)	3 (0.8)	
PR	40 (49.4)	200 (52.9)	
SD	29 (35.8)	121 (32.0)	
PD	3 (3.7)	41 (10.8)	
N/E	8 (9.9)	13 (3.4)	
ORR (CR+PR)	41 (50.6)	203 (53.7)	0.042
DCR (CR+PR+SD)	70 (86.4)	324 (85.7)	0.008

Footnote: CR, complete response; DCR, disease control rate; N/E, not evaluable; ORR, objective response rate; PD, progressive disease; PR, partial response; SD, stable disease.

**Table 2:** Univariable and Multivariable analyses of irAEs (n = 459).

Variables	Univariable analysis		Multivariable analysis	
	OR (95% CI)	*P*	OR (95% CI)	*P*
Age (≥ 60 vs. < 60)	1.47 (0.91-2.39)	0.114		
Gender (female vs. male)	1.14 (0.55-2.19)	0.705		
ICIs treatment cycle	1.06 (1.02-1.12)	0.006	1.06(1.02-1.12)	0.006
Smoking status (former/current vs. never)	1.06 (0.58-1.87)	0.833		
Metastasis (yes vs. no)	1.45 (0.89-2.35)	0.130		
No. of treatment line (≥ 2 vs. 1)	1.30 (0.67-2.71)	0.460		
Preexisting diseases (yes vs. no)	0.85 (0.50-1.42)	0.549		
Histology (LUAD vs.non-LUAD)	1.04 (0.63-1.74)	0.880		
K^+^ (high vs. low)	1.81 (1.08-3.01)	0.023	1.86(1.10-3.11)	0.019

**Table 3:** Univariable and Multivariable analyses of PFS (n = 459).

Variables	Univariable analysis		Multivariable analysis	
	HR (95% CI)	*P*	HR (95% CI)	*P*
Age (≥ 60 vs. < 60)	0.90 (0.50 -1.64)	0.741		
Gender (female vs. male)	0.78 (0.35 -1.75)	0.543		
ICIs treatment cycle	0.89 (0.82 -0.95)	0.001	0.87(0.80-0.93)	<0.001
Smoking status (former/current vs. never)	1.39 (0.62 -3.12)	0.429		
Metastasis (yes vs. no)	3.74 (1.66 -8.41)	0.001	3.78 (1.64 -8.72)	0.002
No. of treatment line (≥ 2 vs. 1)	2.94 (1.57 -5.50)	<0.001	2.23 (1.17 -4.23)	0.015
Preexisting diseases (yes vs. no)	2.27 (1.05 -4.88)	0.037	2.03 (0.89-4.62)	0.091
Histology (LUAD vs.non-LUAD)	1.53 (0.84 -2.76)	0.163		
K^+^ (high vs. low)	0.43 (0.24 -0.79)	0.006	0.52 (0.28 -0.96)	0.036
