# Supplementary material for: Association of Ion Concentration with Immune-Related Adverse Events and Prognosis in Lung Cancer Patients Treated with PD-1/PD-L1 Inhibitors
Source: J Cancer. 2026 Jan 1;17(1):109–16. doi: 10.7150/jca.120666 (PMC12719593; doi:10.7150/jca.120666)
Supplement: Supplementary file 1 — Supplementary table. [file jcav17p0109s1.pdf]

Table S1.Ion concentration and optimal cut-off values for irAES, ORR ,DCR and PFS

Variables	optimal cut-off value			
	irAEs	ORR	DCR	PFS
K^+	3.88	4.1	3.93	3.93
Na^+	142.2	139.1	139.4	140.3
Cl^-	102.5	101.1	101.4	104.3
Ca^{2+}	2.21	2.31	2.46	2.27
PO_4^{3-}	1.00	0.93	1.03	1.02
Mg^{2+}	0.94	0.93	0.87	0.76
